# Association of Multisetting Community Programs and Policies With Child Body Mass Index: The Healthy Communities Study

**DOI:** 10.5888/pcd17.190196

**Published:** 2020-05-07

**Authors:** Vicki L. Collie-Akers, Stephen B. Fawcett, Jerry A. Schultz, Kandace K. Fleming, Rebecca E. Swinburne Romine, Lorrene D. Ritchie, Edward A. Frongillo, S. Sonia Arteaga

**Affiliations:** 1 Department of Population Health, University of Kansas Medical Center, Kansas City, Kansas; 2 Center for Community Health and Development, University of Kansas, Lawrence, Kansas; 3 University of Kansas, Life Span Institute, Lawrence, Kansas; 4 Nutrition Policy Institute, Division of Agriculture and National Resources, University of California, Berkeley, California; 5 Department of Health Promotion, Education, and Behavior, Arnold School of Public Health, University of South Carolina, Columbia, South Carolina; 6 Office of the Director, National Institutes of Health, Bethesda, Maryland

## Abstract

**Introduction:**

Expert opinion suggests that efforts to address childhood obesity should seek to transform the environments in which children operate. The objective of this study was to describe the extent to which multisetting programs and policies interact with community and child predictors and are associated with child body mass index (BMI) in the 130 US communities participating in the Healthy Communities Study.

**Methods:**

For 2 years beginning in fall 2013, we collected data through key informant interviews on community programs and policies related to healthy weight among children that occurred in the 10 years before the interview. We characterized community programs and policies by intensity of efforts and the number of settings in which a program or policy was implemented. Child height and weight were measured during household data collection. We used multilevel modeling to examine associations of community programs and policies in multiple settings and child and community predictors with BMI *z* scores of children.

**Results:**

The mean number of settings in which community policies and programs were implemented was 7.3 per community. Of 130 communities, 31 (23.8%) implemented community programs and policies in multiple settings. Higher-intensity community programs and policies were associated with lower BMI in communities that used multiple settings but not in communities that implemented programs and policies in few settings.

**Conclusion:**

Efforts to prevent childhood obesity may be more effective when community programs and policies are both intensive and are implemented in multiple settings in which children live, learn, and play.

**Summary**

**What is already known on this topic?**

Expert opinion suggests that addressing child obesity across multiple settings, such as schools and community organizations, is important. Little evidence is available on the extent to which communities are engaged in implementing efforts in multiple settings and whether implementation in multiple settings is associated with lower body mass index.

**What is added by this report?**

Examination of community programs and policies implemented in 130 US communities demonstrated that communities in which both intensive and multisetting strategies were implemented had lower child body mass index.

**What are the implications for public health practice?**

Practitioners could strengthen their efforts to address childhood obesity by designing interventions that are both intensive and implemented across multiple settings.

## Introduction

Obesity — including childhood obesity — is a public health challenge in the United States (1) and globally (2). Recommended environmental strategies for obesity prevention at the population level focus on making behaviors related to physical activity, healthy nutrition, and healthy weight easier and more likely (3). For instance, many collaborative partnerships for healthy living aim to alter the environment to increase healthy food and beverage choices and opportunities for physical activity. Such population health strategies seek to modify the environment for engagement in physical activity and healthy nutrition by transforming the multiple settings in which children and adults live, work, and play.

Multisetting, comprehensive intervention strategies are widely regarded as best practice in efforts to improve population health (4,5). Recommendations for accelerating progress in obesity prevention call for comprehensive strategies that focus on transforming environments in multiple relevant settings — including schools, retail outlets, health care, workplaces, and media (3). In addition, from a health equity and social determinants perspective (6), attention should be focused on modifying settings that expose children and their families to healthy nutrition (eg, in retail, by eliminating food deserts and places without adequate access to fresh fruits and vegetables) and opportunities for physical activity (eg, in schools, by expanding time for physical activity). Thus, whether from a population health or health equity perspective, the consistent guidance for creating supports for healthy living is to transform environmental conditions in the multiple settings in which children and families are exposed to food and opportunities for physical activity.

We therefore hypothesized that community efforts that bring about changes (eg, new programs, policies, environmental modifications) in multiple settings such as schools and other relevant sectors and settings — for example, youth organizations and businesses — would be more strongly associated with lower prevalence of higher (and unhealthier) body mass index (BMI) than communities in which efforts focused on a narrower array of settings. Little is known, however, about whether multisetting programs and policies are being implemented and are more effective in achieving progress in obesity prevention than efforts targeted toward a narrower range of settings. This study provides an opportunity to test this hypothesis in the context of the national Healthy Communities Study (HCS) (7). Using a community measurement system (8), we gathered data on nearly 10,000 discrete instances of community programs and policies occurring in 130 US communities and designed to promote healthy weight in children. By further characterizing instances of community programs and policies according to the setting in which they occurred, we differentiated between communities in terms of the number of settings in which interventions were implemented. This study examined the extent to which a multisetting implementation 1) is being used by US communities, 2) interacts with potential community predictors (ie, community socioeconomic status and intensity of intervention) and child predictors (ie, sex and family socioeconomic status) identified in analysis of the primary aims of the HCS (9), and 3) is associated with lower BMI among children in those communities.

## Methods

The HCS (2013–2015) was an observational study that examined the association between community programs and policies related to physical activity or nutrition and the weight status of children in 130 diverse communities across the country (7,9). Researchers from Battelle Memorial Research Institute, the University of California Nutrition Policy Institute, University of Kansas, and University of South Carolina designed and implemented protocols to collect retrospective data about community programs and policies implemented in those 130 communities and retrospective and prospective data about BMI among 4,670 participating children (kindergarten through eighth grade). School liaisons (ie, school personnel) recruited students by distributing information forms to parents at each participating school in each community. Research staff members conducted screenings to identify 1 child in each interested family who returned participation forms. Inclusion criteria were being in grades kindergarten through eighth grade, being ambulatory, attending the participating schools, and living in the community for at least 1 year. Communities, defined as a high school catchment area, were selected by using a combination of random and purposeful selection to be national in scope while including communities with a range of characteristics. High school catchment areas included the elementary and middle schools that fed into a single public high school. Described elsewhere in greater detail (10), the 130 communities were distributed across geographic region, urbanicity, income level, and primary racial and ethnic population groups. Sampling occurred through both a national probability-based sample, which included strata to ensure sampling for diverse racial and ethnic populations, region, urbanicity, and income level, and the identification of a sample of communities known to have promising community programs and policies (7). Battelle Memorial Institute’s institutional review board provided oversight (Federalwide Assurance no. 4696). HCS had an appointed observational study monitoring board. The Office of Management and Budget (OMB no. 0925–0649) provided approval.

**Protocol for measuring instances and intensity of community programs and policies.** Fawcett and colleagues (8) described the data collection protocol for community programs and policies. Staff members collected data on community programs and policies from fall 2013 through fall 2015. We used a rolling 10-year period for the inquiry. For example, if data from a particular community were collected in 2014, staff members documented community programs and policies occurring in the 10 years before 2014. Using a field-tested protocol and structured interview, staff members conducted key informant interviews of community members (n = 1,451) representing various sectors (eg, schools, local government, nonprofit organizations). The structured interview included probes to 1) identify community programs and policies known to the key informants, 2) code each discrete community program and policy, and 3) characterize each community program and policy by various attributes, such as goal addressed (eg, physical activity, healthy nutrition) and type of implementation used. In addition to a narrative description of each community program and policy, staff members asked key informants to identify beginning and end dates of implementation, goal(s) addressed (eg, physical activity, healthy nutrition), behavioral change strategies used (eg, providing information; enhancing services and support; changing consequences; modifying access, opportunities, and barriers), duration or frequency of implementation (eg, one-time event, more than once, continuous), and number or proportion of children in the community reached through implementation. In 2016, staff members analyzed information from key informant interviews and other documents to code instances of community programs and policies as meeting explicit criteria, including 1) being a program, policy, or environmental change; 2) occurring in the community (ie, high school catchment area); 3) addressing healthy eating, physical activity, or healthy weight; 4) targeting children aged 4 to 15 years; and 5) occurring in the 10 years before the key informant interview.

Available information was also used to characterize each community program and policy by additional variables, including the setting or sector in which the community program and policy occurred (eg, schools, parks), target of the community program and policy (eg, children in the community, parents/caregivers), and behavioral objectives (eg, increase consumption of fruits and vegetables, decrease consumption of sugar-sweetened beverages, increase engagement in afterschool physical activity). Variables describing duration, reach, and behavioral change strategies were combined to create an index of intensity, the Community Program and Policy Intensity Score (8,11,12). Quality assurance and control procedures were implemented throughout all phases of data collection, coding, and analysis. Quality assurance included calculation of inter-observer agreement for all variables requiring coding, and a minimum of 80% inter-observer agreement was maintained. A total of 9,681 community programs and policies were identified in the selected 130 communities. A previous study described the prevalence and attributes of these programs and policies (13).

**Construction of multisetting variables.** Each community program and policy was further characterized according to the setting in which it occurred. The types of settings characterized in this study were businesses, childcare or preschool sites, community organizations, criminal justice organizations, faith-based organizations, food retailers, health care organizations, family homes, local health departments, state health departments, media outlets, neighborhoods, other government organizations (eg, municipal planning departments), parks and recreation, schools, transportation, youth organizations, and other.

To examine the relationship between community programs and policies implemented across multiple settings, a new variable for each community was created that characterized the distribution of settings in which all community programs and policies occurred in each community. *Multisetting* was defined as having 3 or more settings with at least 20% of community programs and policies in each across all 10 years, or 4 or more settings with greater than 10% of community programs and policies in each. For example, a community with 25% of its community programs and policies in schools, 22% in youth-serving organizations, and 21% in parks would be coded as multisetting. Although the public health literature refers to the term *multisetting*, we found no operational definition that could be used with these data. Decisions on how to define *multisetting* for this analysis were based on the distribution of the data and the need to ensure adequate rather than superficial intervention representation across settings. Communities with either concentrations of community programs and policies in single settings (eg, a concentration of ≥60% community programs and policies for a community occurring in a single setting) or distribution of community programs and policies across many sectors with few community programs and policies in each category were scored as *nonmultisetting*. For instance, a community in which 61% of its community programs and policies occur in schools would be coded as nonmultisetting because a single setting dominates. Another example of nonmultisetting is a community in which community programs and policies are distributed across 8 sectors, but 6 of those sectors have only 1 community program and policy.

**Collection of BMI data.** Trained study staff members conducted home visits to collect data for calculating BMI (14). Staff members measured height and weight for each child by using calibrated scales and portable stadiometers. Each measure (height and weight) was taken twice and entered immediately into the study’s information management system. If disagreement between the 2 instances of measurement occurred, the information management system required a third measure. We calculated BMI as weight in kilograms divided by height in meters squared (kg/m^2^). A total of 4,670 children aged 4 to 15 years had at least 1 BMI measurement (Table 1).

**Table 1:** Demographic Characteristics of Children (N = 4,670) Participating in the Healthy Communities Study, 2013–2015^a^

Characteristic	No. (%)
**Age, y**	
4	33 (0.7)
5	269 (5.8)
6	547 (11.7)
7	618 (13.2)
8	526 (11.3)
9	501 (10.7)
10	487 (10.4)
11	504 (10.8)
12	495 (10.6)
13	464 (9.9)
14	209 (4.5)
15	17 (0.4)
**Sex**	
Male	2,303 (49.3)
Female	2,367 (50.7)
**Race^b^**	
African American	854 (21.1)
American Indian/Alaska Native	55 (1.4)
Asian	151 (3.7)
Native Hawaiian/Pacific Islander	8 (0.2)
White	2,767 (68.4)
More than one race	210 (5.2)
**Ethnicity^c^**	
Hispanic	2061 (44.5)
Non-Hispanic	2570 (55.5)
**Annual family income level, $**	
<20,000	1,247 (26.7)
20,000–35,000	1,098 (23.5)
35,000–50,000	595 (12.7)
50,000–75,000	514 (11.0)
75,000–100,000	381 (8.2)
>100,000	835 (17.9)

a The Healthy Communities Study was an observational study that examined the association between community programs and policies related to physical activity or nutrition and the weight status of children in 130 diverse communities in the United States (7,9). Data were collected from fall 2013 through fall 2015; 4,670 children aged 4 to 15 years had at least 1 measurement of height and weight for the calculation of body mass index.

b Race was not reported for 625 children.

c Ethnicity was not reported for 39 children.

**Analysis.** We used multilevel modeling to analyze relationships between child BMI, the intensity of community programs and policies, and a multisetting implementation of an obesity intervention. We examined a series of multilevel models using SAS version 9.4 (SAS Institute Inc) and the SAS MIXED procedure, in which 4,670 children were nested within 130 communities. We used maximum likelihood estimation to estimate and report all model parameters. We evaluated the significance of independent variables (ie, fixed effects) by using *z* tests of the ratio of each estimate to its standard error. Community variation (ie, random effects) was evaluated by using likelihood ratio tests. We evaluated effect size by using pseudo-*R* ^2^ values for the proportion reduction in each variance component. To facilitate interpretation of effects, we grand-mean–centered predictors so that the effect of each predictor can be interpreted as the effect for someone scoring at the mean on the other predictors in the model. Child BMI *z* scores differed significantly across communities and were modeled by using random intercepts. The relationship between child BMI *z* score and child sex and between child BMI *z* score and family income did not differ significantly across communities, so we did not model random slopes for child predictors, and we evaluated the predictors by using fixed effects only. Child predictors of sex and family income were first entered as fixed effects into the model, followed by the community predictors of intensity, community graduation rate, and a dichotomous indicator for a multisetting implementation as well as interactions with it. We chose child and community predictors on the basis of a previous analysis in which these factors were identified as being associated with BMI (9). We conducted our analysis in October 2018.

## Results

Of the various types of settings in which community programs and policies were implemented during the study period, education/schools was the most frequent setting (44.0% of community programs and policies), followed by youth-serving organizations (19.6%) and parks and recreation (17.4%). The community/neighborhood setting was identified in 7.6% of community programs and policies; childcare organizations, faith-based organizations, government organizations (public health and other), health care, media, private sector, transportation, and “other” had less than 2.5% of community programs and policies in each category. Across all 130 communities, the mean number of settings during the 10-year period in which community programs and policies were implemented was 7.3 (standard deviation, 1.6; range, 3–12). Sixteen (12.3%) communities reported having community programs and policies in 5 or fewer settings, whereas 28 (21.5%) communities reported having community programs and policies in 9 or more settings.

**Figure:**
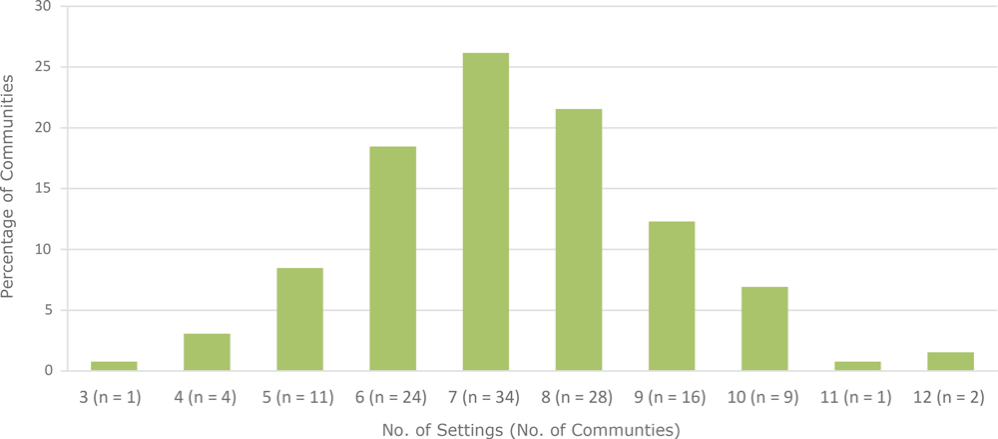
Distribution of communities (N = 130) participating in the Health Communities Study, by number of settings in which communities implemented community programs and policies.

**Table:** 

Number of Settings (Number of Communities)	Percentage of Communities
3 (n = 1)	0.8
4 (n = 4)	3.1
5 (n = 11)	8.5
6 (n = 24)	18.5
7 (n = 34)	26.2
8 (n = 28)	21.5
9 (n = 16)	12.3
10 (n = 9)	6.9
11 (n = 1)	0.8
12 (n = 2)	1.5

Of the 130 communities, 23.8% (n = 31) met the criteria for having multisetting implementation. The remaining 76.2% (n = 99) of communities were identified as nonmultisetting. Twenty-seven of these 99 communities were dominated (≥60% of community programs and policies) by community programs and policies that occurred in a single setting. The other 72 communities had community programs and policies in multiple settings with low numbers of community programs and policies in most settings.

The intraclass correlation of child BMI *z* scores, as predicted by a null (ie, no predictor) random-intercept model, was 0.06, indicating that 6% of the variance in BMI was among communities. A 95% random-effects confidence interval showed that 95% of the sample communities were predicted to have average BMI *z* scores from 0.15 to 1.25. The average community BMI z score for girls with an average family income in a community with an average high school graduation rate, an average-intensity intervention (ie, a community with a community program and policy intensity score at the mean), and a multisetting intervention implementation was 0.74; the corresponding average BMI *z* score for boys was 0.81.

In the final model with both child and community predictors, we found a significant effect for sex: boys had a significantly higher BMI z score than girls, by 0.07 (Table 2). Child family income was also a significant predictor of BMI z scores: for every 1-point increase in the average family income score of 3.04, the predicted BMI *z* score decreased by 0.077. At the community level, the higher the community graduation rates, the lower the community average BMI z scores. Additionally, we found a significant interaction between community graduation rate and child family income such that for every 1-point increase in a community’s graduation rate, the decrease of 0.077 in predicted BMI *z *score became a decrease of 0.477 for every 1-point increase in family income.

**Table 2:** Fixed and Random-Effect Estimates From Final Multilevel Model Predicting Child BMI *z* Scores Among Children (N = 4,670) Participating in the Healthy Communities Study, 2013–2015^a^

Characteristic	Estimated BMI z Score	Standard Error	95% Confidence Interval	*P* Value
**Fixed effects**				
Intercept	0.742	0.051	0.641 to 0.842	.001
Child sex is male	0.070	0.030	0.004 to 0.137	.04
Child family income, grand mean centered	−0.077	0.011	−0.098 to −0.054	<.001
Community graduation rate, grand mean centered	−1.094	0.223	−1.537 to −0.651	<.001
Intensity of community programs and policies,^b^ grand mean centered	−0.421	0.233	−0.881 to 0.038	.07
Multisetting = 0	−0.054	0.054	−0.160 to 0.052	.32
Intensity of community programs and policies^b^ × multisetting = 0 interaction	0.581	0.268	0.051 to 1.110	.03
Community graduation rate × child family income interaction	−0.400	0.105	−0.605 to −0.195	<.001
**Random effects**				
Intercept variance	0.020	0.008	—	.008
Residual	1.335	0.028	—	<.001

Abbreviation: BMI, body mass index.

a The Healthy Communities Study was an observational study that examined the association between community programs and policies related to physical activity or nutrition and the weight status of children in 130 diverse communities in the United States (7,9). Data were collected from fall 2013 through fall 2015.

b Intensity of community programs and policies refers to the individual scores assigned to each community program and policy based on characterization of the reach, duration, and strategy. These individual scores were aggregated annually for each community to create an annual intensity score.

We found a significant interaction between intensity and multisetting intervention implementation (*F*_1,121_ = 4.71; *P* = .03) (Table 3), indicating that the difference in the influence of intensity (0.581) between multisetting communities and nonmultisetting communities was significant. Intensity of community programs and policies was negatively related to BMI *z* score in communities that used multiple settings; the higher the intensity, the lower the BMI *z* score. For example, in a multiple-setting community with an intensity score 1.0 higher than the mean, the predicted BMI z score was expected to be 0.42 lower. BMI *z* score was positively but not significantly related to intensity of community programs and policies in communities that did not use multiple settings. For example, in a community with an intensity score 1.0 higher than the mean, the predicted BMI z score was expected to be 0.16 higher.

**Table 3:** Test of Fixed Effects From Final Model Predicting BMI *z* Scores Among Children (N = 4,670) Participating in the Healthy Communities Study, 2013-2015^a^

Effect	*F* Value*_df_*	*P* Value
Child sex	4.30_1,4645_	.04
Community graduation rate, grand mean centered	24.08_1,84.1_	<.001
Child family income, grand mean centered	47.82_1,3526_	<.001
Intensity of community programs and policies,^b^ grand mean centered	0.96_1,121_	.33
Multisetting	1.01_1,98.8_	.32
Intensity of community programs and policies^b^ × multisetting	4.71_1,121_	.03
Community graduation rate × child family income	14.63_1,1575_	<.001

Abbreviation: BMI, body mass index.

a The Healthy Communities Study was an observational study that examined the association between community programs and policies related to physical activity or nutrition and the weight status of children in 130 diverse communities in the United States (7,9). Data were collected from fall 2013 through fall 2015.

b Intensity of community programs and policies refers to the individual scores assigned to each community program and policy based on characterization of the reach, duration, and strategy. These individual scores were aggregated annually for each community to create an annual intensity score.

## Discussion

The HSC provided the opportunity to expand understanding of how communities are working to address childhood obesity and how these efforts are related to child BMI. Data from the HCS suggest that implementation of community programs and policies across multiple settings is relatively common, with the average number of settings in which community programs and policies were implemented being 7.3 across the 130 study communities. For nearly a quarter of communities, efforts were focused on a single setting, most commonly schools, whereas nearly a quarter of communities used what we categorized as a multisetting implementation.

This analysis did not yield a significant main effect for a multisetting implementation or intervention intensity on community average child BMI *z* scores, but we found a significant interaction between the two. This analysis did not yield a significant main effect for intervention intensity on community average child BMI *z* scores, which was found longitudinally in another study (9), but our finding was consistent with findings in another cross-sectional study (15), in which intensity was a nonsignificant predictor. Our analysis found a significant interaction between a multisetting implementation and intervention intensity. For communities with a multisetting implementation, higher intensity of community programs and policies tended to be associated with lower BMI *z* scores, whereas for communities without a multisetting implementation, higher intensity was associated with higher BMI *z* scores. These findings suggest that the health of children is improved when community programs and policies are sufficiently intense and are diffused across many settings in which children interact. Many factors may explain this finding, including that if a higher intensity set of community programs and policies is concentrated in only one or a few settings, the other settings with fewer health-promoting programs and policies may be ones in which children spend more time or may be exploited more heavily for unhealthy eating and sedentary behavior.

Few studies have systematically examined associations between community interventions implemented across multiple settings and an important outcome such as child BMI. The large sample of 130 communities and nearly 5,000 children and extensive data collection efforts for nearly 10,000 community programs and policies — and their characterization by attributes related to intensity (reach, duration, strength) and multisetting distribution — permit analyses of associations rarely examined.

This observational study can examine associations, but it cannot establish a causal relationship. In a retrospective study, community program and policy data may be influenced by key informants’ abilities to recall existence or details of what was implemented. Although we took care to seek key informants from different types of settings, informants in some communities may have focused on a narrower (or broader) range of settings. In addition, the reporting of community programs and policies may have been influenced by the extent to which key informants were identified across the settings.

This finding has important implications for practitioners and researchers, suggesting that planning for interventions to prevent childhood obesity should pair 1) a sufficient number of programs and policies of higher intensity (ie, longer duration, fuller reach, and greater strength of strategies) and 2) implementation among multiple settings through which children can be exposed to these interventions. In addition, it suggests caution in attempting to diffuse across multiple settings if resources and strategies are not available to support sufficient intensity. The analysis included in this study also identified associations between BMI *z* scores and community-level social determinants of health such as income and education. Additional research and practice are needed to understand how to develop and implement community programs and policies with sufficient intensity and diffusion across settings to ensure benefit for all children and promote equity. Community guidelines for comprehensive preventive interventions might include recommendations for implementing intensive community programs and policies (9), targeting of multiple behaviors related to the goal (15), and intervening in multiple settings.

Further research can help expand our understanding of what dose and delivery of preventive interventions are needed to improve population health outcomes (16). Previously, expert opinion has held that community programs and policies should be of strong dose and targeted delivery. This study adds to that knowledge base and recommends an intensive dose of community programs and policies delivered through those multiple settings in which people live, learn, work, and play.
